# The Diverse Range of Possible Cell Membrane Interactions with Substrates: Drug Delivery, Interfaces and Mobility

**DOI:** 10.3390/molecules22122197

**Published:** 2017-12-11

**Authors:** Hyun-Sook Jang

**Affiliations:** Center for Soft and Living Matter, Institute for Basic Science (IBS), Ulsan 44919, Korea; hs84.jang@gmail.com; Tel.: +82-10-3859-9275

**Keywords:** bio-membrane, nanocarriers, bio-interfaces, mobility, nanoparticles

## Abstract

The cell membrane has gained significant attention as a platform for the development of bio-inspired nanodevices due to its immune-evasive functionalities and copious bio-analogs. This review will examine several uses of cell membranes such as (i) therapeutic delivery carriers with or without substrates (i.e., nanoparticles and artificial polymers) that have enhanced efficiency regarding copious cargo loading and controlled release, (ii) exploiting nano-bio interfaces in membrane-coated particles from the macro- to the nanoscales, which would help resolve the biomedical issues involved in biological interfacing in the body, and (iii) its effects on the mobility of bio-moieties such as lipids and/or proteins in cell membranes, as discussed from a biophysical perspective. We anticipate that this review will influence both the development of novel anti-phagocytic delivery cargo and address biophysical problems in soft and complex cell membrane.

## 1. Current Stages of Membranes World in Drug Carriers 

**Figure 1:**
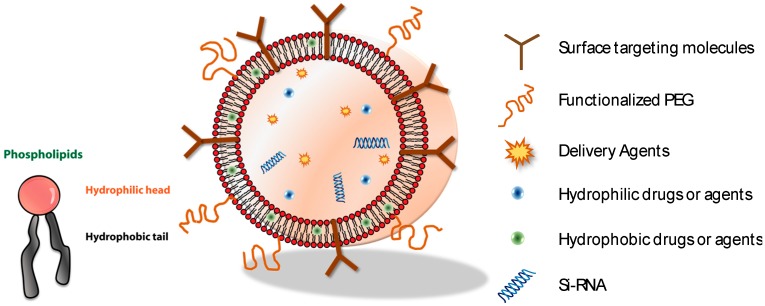
The schematic of the self-assembly of a core-shell structure (i.e., vesicles) consists of amphiphilic molecules and a variety of biological moieties and agents. One of the representative amphiphilic molecules, phospholipids, is composed of hydrophilic heads and hydrophobic tails.

Cell membranes are highly complex dynamic systems with compositional heterogeneity that utilize several types of phospholipids and proteins as major constituents. The cell membrane contains a self-assembling nature that results in bilayered structures a few nanometers thick. In addition, it is responsive to external stimuli such as chemical (i.e., ion, concentration) [1,2,3], mechanical forces (i.e., pressure) [4,5,6], electrical (i.e., voltage) [4,7,8,9], and thermal stresses (i.e., temperature, light) [10,11], and functions by communicating with other cell membranes and transporting signals in a harmonious manner without the disrupting of the membrane. Motivated by the self-signaling functions of cell membranes, mimicking the cell membrane has gained attention as a method of developing nanodevices in drug delivery, diagnosis, and soft-robotics [12,13,14,15,16]. One of the most well-known methods for developing an artificial cell membrane is a bottom-up technique in which different moieties with various charges, molecules, and functional groups are self-assembled via noncovalent bonding or chemical conjugation [17,18,19,20]. For self-assembly purposes, molecules should include an amphiphilic nature, in which both hydrophilic and hydrophobic parts are present. Figure 1 shows representative amphiphilic molecules, phospholipids, which have a hydrophilic head and a hydrophobic tail, and its assembled morphologies (i.e., vesicles) with various loading cargo. They can adopt multiple meso-phases depending on their molecular structures [18,21] and perform self-assembly in nanoscales. The vesicle comprises core materials surrounded by bilayered amphiphilic molecules. As the chemical natures of the core (i.e., fluid or organic/inorganic materials) and shell (i.e., phospholipids) in the vesicle are different, the structure of vesicle is termed as core-shell structure [22,23]. In addition, amphiphilic molecules such as lipids (i.e., phosphatidylcholine and phosphatidylethanolamine) have low toxicity and good biocompatibility [24]. Thus, various morphologies composed of amphiphilic molecules, such as vesicles [19,25] and disks [18,21,26,27,28], have taken center stage as pharmaceutical loading cargos for drugs [29,30], genes [30,31], and DNA [32,33]. However, the bio-applicability of these nano-carriers faces various biological barriers in our immune system, such as reticuloendothelial system (RES) effects [12], shape transformation during phagocytic internalization [34], and other degradation factors caused by protein absorption [35]. Nevertheless, the Janus properties of amphiphilic molecules, which allow hydrophobic layers to overcome the loading barriers of drugs and inorganic particles, while hydrophilic layers act as a decoy for surface moieties (i.e., PEG [36], aptamer [37], and proteins [38]) will help increase biological functionality (i.e., targeting, body circulating retention time) and eventually increase the therapeutic efficiency.

**Figure 2:**
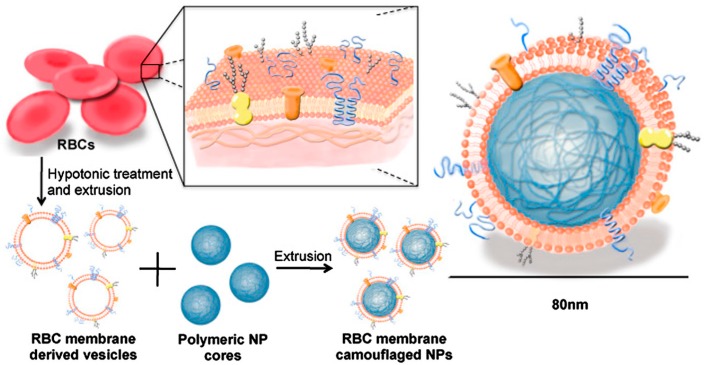
The schematics for the preparation process for RBC-membrane-coated PLGA NPs. RBC membrane-coated NPs are prepared using an extrusion-based fusion process between the osmotic shock-derived RBC membranes and the nano-sized PLGA particles. Adapted with permission from [39] copyright 2011, Proceedings of the National Academy of Sciences.

Recently, top-down approaches to develop a biomimetic drug delivery cargo have resulted in hybrid nanoparticles (NPs) made of biodegradable polymeric NPs and native cell membranes. It has gained lots of attention because they have cell-like functions and copious immune-compatibility due to the intact proteins in their extracellular membranes [39,40,41,42,43]. The benefit of using genuine extracted membranes in preparing hybrid NPs is that the sophisticated molecular design (i.e., stoichiometry, charge ratio, and sample preparations) with the guaranteed morphologies (i.e., vesicle, rod, etc.) that are required in the bottom-up technique can be reduced. Note that the native membranes comprising various types of phospholipids and membrane-associated proteins still have amphiphilic properties, implying that the self-assembling tendency into “bilayers” is guaranteed in aqueous solutions. In addition, the extracted cell membrane has a camouflage function in the body due to being the same protein, meaning that the body’s immune system easily accepts the foreign molecules (i.e., drugs in hybrid-NPs). Hu et al. [39] first introduced RBC membrane-coated poly (lactic-co-glycolic acid) (PLGA) NPs. This platform is mainly divided into two parts: (i) derivation of cell membrane-vesicles from the desired cells and (ii) fusion of the ghost membrane vesicle with nanoparticles by extrusion or sonication (Figure 2). The cell membranes are isolated by hypotonic lysis and multiple steps of centrifugation to remove intracellular components. The emptied RBC ghost membranes vesicles can be obtained using sonication or extrusion. Finally, the RBC membrane-derived vesicles are fused with PLGA NPs through external forces such as extrusion or sonication, which gives the hybrid NPs immune evasion properties with native cell membrane coatings. Specifically, the surface proteins of CD 47 in RBCs behave as self-markers responsible for immune evasion, which prevents them from macrophage uptake [44,45,46,47,48]. These externally decorated NPs (with native cell membranes) both increase the bioavailability of encapsulated drugs and lead to a higher surface area to volume ratio in nanoscales, allowing for high drug loading and reactivity in the environment. Consequently, it was shown that cell membrane-coated NPs prolong body retention times with an increased probability of accumulation in tumors, improved site-specific binding, and enhanced therapeutic efficacy with low toxicity [41,49]. Since the compositions of lipids and proteins vary by the source and determine cellular functions such as immunological impact, there have been attempts to coat biodegradable PLGA NPs with various types of cell membranes such as those of eukaryotic RBCs [39], cancer cells [42], platelets [43], leukocytes [50], and bacteria [40,51], as presented in Table 1. Furthermore, a smart concept for hybrid-NPs was developed by adding a self-driving force since the incorporated NPs can be stimulated by various sources, such as magnetic fields [51,52], acoustic fields [53,54,55], electric fields [56,57], light [58,59], or chemical fuels [10,54,60] while upholding their bioavailability through their surface-anchored proteins. The control of the speed, directionality and temporal behavior of such nanomotors still needs to be investigated for the development of hybrid-NPs from native membranes.

## 2. Nano-Bio Interfaces between Cell Membranes and Particles

**Figure 3:**
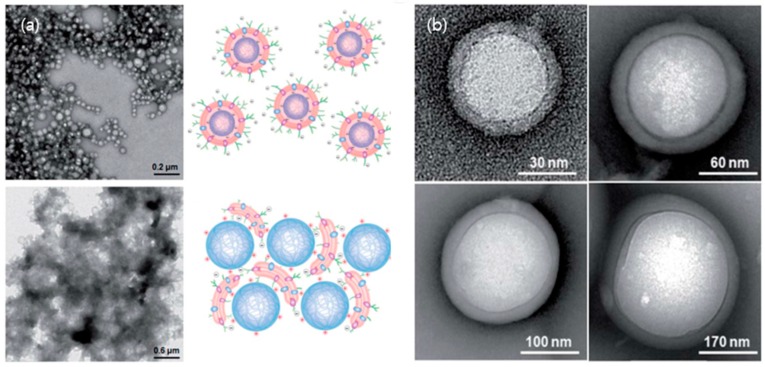
(**a**) The effect of surface charge of particles on RBC membrane coating. The upper left corner shows a TEM image of negatively charged polymeric particles extruded from RBC membranes while the upper right corner illustrates the electrostatic interaction between negatively and asymmetrically charged RBC membranes with negatively charged polymeric cores. The opposite case, positively charged polymeric cores with RBC membranes is shown in a TEM image (bottom left) as well as its possible electrostatic interactions (bottom right); (**b**) The effect of substrate particle curvature induced by particle sizes on RBC membrane coating. Representative TEM images show RBC-NPs with a variety of NP cores sized 65, 120, 200, and 340 nm in diameters, indicating uniform RBC cloaking on various sizes of NPs. Adapted with permission from [90] copyright 2011, Royal Society of Chemistry, Nanoscales.

**Figure 4:**
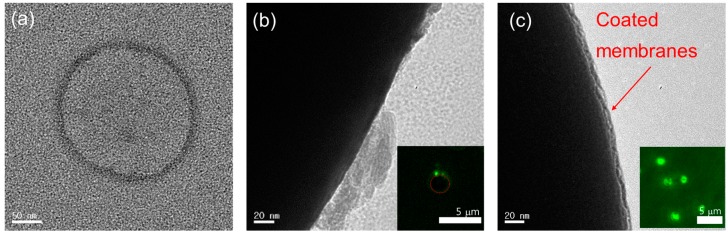
TEM images with uranyl acetate negative staining that depict (**a**) RBC membrane-derived vesicles without the core substrates after sonication, (**b**) a low surface cover ratio of RBC membranes to Si particles (2 μm), which shows the partial debris of membranes on substrates, and (**c**) a high surface cover ratio of RBC membranes to Si particles with the successful translocation of the membrane onto the Si particles. The inset figures show a confocal image of RBC-membrane-coated hybrid Si particles with the low and high surface cover ratio of RBC membranes to particles in the phosphate buffered saline solution. The membrane was labeled with fluorescence lipids (DMPE-RhB) of 2 μM.

In addition to the use of the cell-membrane-coated NPs in drug delivery, exploiting various bio-interfaces within the membrane-particle assembly is highly achievable because the selection of NPs’ physical properties is tremendous, including with different sizes [79], elements [80,81], shapes [82,83,84] and surface charges [85,86]. In addition, the cell membrane itself contains inherited heterogeneities with various types of proteins and phospholipids, yet in an arranged manner and with nanoscale thicknesses. For instance, the plasma membrane proteins in the cell face both the interior and the extracellular fluid of the cell, which surrounds all cells. In the case of RBCs, the surface glycans (glycocalyx), which are responsible for the stabilization and immune-evasive properties of the cell, are distributed asymmetrically in the extracellular side of RBCs and result in charge asymmetry across cellular membranes due to the abundant negatively charged sialyl residues at the glycan terminus [87,88,89]. Therefore, cell membranes contain various interfaces that may arise in asymmetricity in the bilayered membranes, and the conformation of proteins depends on the surrounding environment (i.e., hydrophilicity). Recall the formation procedure for the RBC membrane-NP assembly mentioned above; the fusion processes between the cell-derived membrane vesicles and PLGA NPs were present under sonication. Deciphering how the interfaces of the native membrane and NPs interact during the membrane-fusion processes may benefit us not only in designing a successful membrane–particle assembly, but also in understanding physical chemistry and colloidal physics. Luk et al. [90] have investigated the aspects of interfacial interactions between natural cellular membranes and polymeric NPs as substrates. They examined the effects of various properties of PLGA NPs, such as surface charges, surface curvatures in the range of 65–340 nm, the completeness of membrane coverage and the effect of membrane sidedness upon the coating during the membrane cloaking [90]. Their study showed that the negatively charged RBC membranes completely covered the negatively charged polymeric NPs, which have a surface potential of approximately—45 mV with various curvatures, in a right-side-out manner, resulting in core-shell NPs structures (upper left in Figure 3a). In addition, different substrates such as silica, gelatin, and gold particles were successfully exploited to obtain the hybrid-NPs with RBC membranes [49,91]. Nevertheless, in the case of the positively charged PLGA NPs with a coated layer of polyethylenimine (PEI), the homogenous coating of the membrane onto the NPs failed and aggregated by bridging each other in places where the surface potential is about +25 mV (bottom left in Figure 3a). It suggests that the successful coverage of RBC membranes on the substrate of NPs requires moderate affinity (which will likely vary by cell type), and allows certain mobility and local rearrangements of the extracted membranes along the nanoscale colloidal surfaces. In fact, it is known that the extracellular membranes contain more innate, dense and negatively charged sialyl moieties with small domains [92]. Therefore, a strong charge affinity between bio-membrane and colloidal particles makes them collapse onto the large aggregates instead of forming core-shell NP structures (upper and bottom right in Figure 3a). In addition, the flexibility of the RBC membrane coating for the core-shell NPs structure has been examined with different sizes of negatively charged PLGA particles cores (diameters, 65, 120, 200, and 340 nm; Figure 3b). The transmission electron microscopy (TEM) images showed uniform RBC membrane cloaks on PLGA NPs resulting in core-shell NPs structures of diameter ≤340 nm. These results open the possibilities of functionalizing a wide range of nanodevices with different sizes for specific medical applications. Besides the sizes, charges of NPs, and other factors that govern the RBC membrane cloaking on NPs are shown in Figure 4. The structures and the interfaces of the assembly were confirmed by TEM (JEOL JEM-1400) at 100 kV. The samples were dispersed on holey carbon grids and then fully dried for the observation. In addition, to increase the contrast of the biological samples under TEM, the typical method of negative staining with a heavy atom, 1 wt. % of uranyl acetate solution to sit on the surface of the membranes, has been applied. As any fluids in the TEM grids will be eliminated during the drying process, the negatively stained membrane edge or materials of a high electron density will appear in the images. Figure 4a shows the emptied vesicle morphology made of the extracted RBC membrane after the extraction and sonication under TEM. In addition, the emptied cellular content but intact cellular structure in RBC after hemolytic treatment in hypotonic solution has been previously reported [39]. Another interesting point is that while the fusion of the RBC membrane-derived ghost vesicles (without NPs) yielded a polydispersity in sizes upon sonication, the RBC-derived PLGA NPs exhibited unimodal distributions and increased the size (approximately 10 nm) compared to the bare NPs [39]. This indicated that the core-NPs behave as nucleated seeds and aid in yielding the uniform size of the hybrid-NPs’ core-shell structures. Moreover, it has been reported that the surface coverage ratio of particles to the native membrane plays a key role in ensuring the uniform membrane coating over the NP substrates [90]. This review further confirms the effects of the surface cover ratio of particles to RBC membranes by a substrate of Si particles with a size of 2 μm and a surface potential of −44 ± 0.7 mV as measured by the zeta potential (Zetasizer Nano ZS, Malvern Instruments, Malvern, UK) with a 633 nm red laser. It has been reported that the relative ratio of the particles to membranes can be adjusted by the size of particles at a given concentration and the total surface area of the hybrid-NPs (S_total_) [90]. Afterwards, the required RBC membrane (V_mem_) volume for coating the colloidal particles can be established with the following Equation (1):

V_mem_ = S_total_/average surface area of the cell/concentration of the cell (1)

Two parameters, a low surface coverage (50% of the required V_mem_) and a high coverage ratio (100% of the required V_mem_), are considered to confirm the effect of the relative ratio of particles to the membrane on the formation of the membrane-particle assembly. While the dark features in Figure 4b,c represent the Si particles because the high electron density from high atomic number provides the high contrast in TEM, the bright aggregates or layer on the Si particles are negatively stained extracted membranes. As depicted in Figure 4b,c, while a low surface coverage ratio of the membrane to particles resulted in a fragmented coating of the RBC membrane on the Si particle, the successful translocation of RBC membranes onto the surface of Si particles with uniform layers was observed at a high coverage ratio. Furthermore, the completeness of the membrane coating was confirmed by the confocal microscopy (Leica TCS SP8 STED, Leica Microsystems) after labeling dyes of 1,2-dimyristoyl-sn-glycero-3-phosphoethanolamine-*N*-lissamine rhodamine B sulfonyl dyes (DMPE-RhB) in the RBC membrane with 2 μM as shown in inset Figure 4b,c and in Video S1 and S2 in the Supporting Information. Accordingly, the key factor for designing hybrid NPs with empowered immune compatibility was controlling the electrostatic force using surface charges and the right surface cover ratios. Future work may be performed to investigate how the cell membrane interactions that happened at other bio-interfaces, such as protein-protein or DNA-protein interactions and enzyme-triggered destabilizations, can locally perturb other functionality along the colloidal surface.

## 3. Mobility of the Lipid-Protein Complexes in Native Membranes

**Figure 5:**
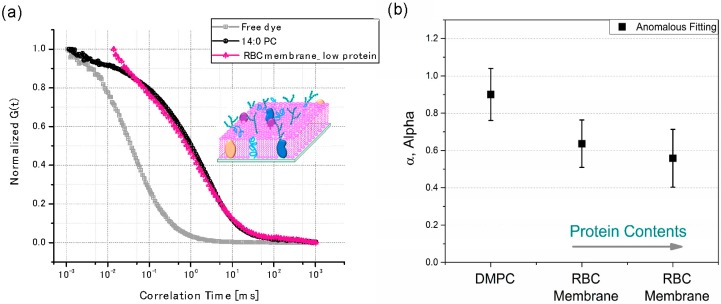
(**a**) The FCS curves of three different systems. Free dye (Rh6G) is in gray, supported lipid bilayers (SLB) composed of the DMPC are in black and extracted ghost RBC membranes are in the red labeled with DMPE-RhB (2 μM); (**b**) The effect of the total protein contents on the sub-diffusiveness (*α*) of supported RBC membranes. The protein contents were confirmed with a bicinchoninic acid assay (BCA).

The mobility of membrane physics is intriguing in that collective diffusion can replace the independent diffusion of individual molecules [93,94,95]. In addition, the mobility of molecules in the cell membranes is closely related to its functions. In the case of RBCs, the functions of cholesterol and sphingolipid-rich membrane domains, which are also called lipid rafts, are very important for improving signal transduction [96]. The sizes of these domains vary from micro- to nano-meters depending on the type of cells, and the mobility of membranes changes locally due to the inhomogeneous distribution of these domains in the lipid membranes [97,98,99,100]. The sub-populations of domains with lipid and membrane-associated proteins are caused by different spatial compositions and components in the membranes, keeping the limited miscibility or the altered local membranes stiffness and fluidity [97,101,102,103]. In addition, the interplay of lipid membrane-mediated protein assembly in domains such as proteolipid complexes significantly perturbs the mobility and functionality of such membranes [97,98,99,100]. Consequently, membrane-coated NPs also interact with the sub-domains that are responsible for the membrane association of proteins, ligand-receptor binding, and membrane-bound protein-protein interactions. It is known that biological processes such as the adsorption of peripheral membrane proteins to the outside of a RBC cell not only affect the mobility of lipids where proteins reside but also of those on the other leaflet (i.e., on the cytosolic side). This resulted in inhomogeneous protein distributions and further influenced membrane-mediated cell functions such as docking [104] and formation of synapse [1,5,104,105]. In addition, a recent study shows that envelope glycoprotein mobility on HIV-1 particles can dictate the maturation state of the virus [106]. A variety of techniques such as a single particle tracking [107,108], fluorescence resonance energy transfer (FRET) [109], and fluorescence correlation spectroscopy (FCS) have been applied to observe how dynamic molecular functions work in cell membrane (i.e., lipid rafts) [94,97]. However, the domains have relatively short lifetimes, and observing interactions in intact cells is challenging. Here, FCS is a unique technique that measures the dynamic movement of the sub-populated molecules in membrane systems (i.e., phospholipids) using a highly sensitive and non-invasive technique with a good spatial resolution [94,97,110,111]. Diffusion coefficients (D) are determined from the transit time ($\tau_{D}$) of fluorescent tagged molecules in a very small observation volume (less than 10^−15^ L) using a confocal setup. Many studies have measured the physical properties of the domains in the biological membranes by focusing on the development of artificial bio-membranes using either supported lipid bilayers (SLB) with Langmuir-Blodgett films or a vesicle disruption approach [112,113]. These protocols are mostly based on bottom-up techniques that frequently require multiple steps for each process, such as solvent evaporation, the optimization of the molar ratio between lipids and proteins for the formation of the domains, and the optimization of ionic strength and/or size; they also required an extremely clean environment. The native membrane may be a good candidate to overcome these barriers as it still contains most of the compartments within the sub-domains, their innate functionalities, and the self-assembled bilayers. In addition, no further modification is required to form the SLBs on the glass substrates. In this review, the preparation of SLBs was achieved by a fusion through adsorbed the membrane-derived vesicles of 100 nm on the glass substrates under an ionic strength (i.e., 50–100 mM), as reported in the literatures [113,114,115]. The mobility of the phospholipids in the RBC membranes has been confirmed with FCS techniques (Leica TCS SP8 STED, Leica Microsystems). Figure 5a shows the FCS curves of rhodamine 6G free dye, two different SLBs made of 1,2-dimyristoyl-sn-glycerol-3-phosphocholine (DMPC) and the RBC ghost cells. The membranes were labeled with 2 μM of a DMPE-RhB fluorescence dye. The FCS analysis in this review is based on the fluorescent-labeled phospholipid analog molecules (DMPE-RhB) diffusing laterally in SLBs. Under the geometry of the confocal microscopy, the time traces of the fluorescent bursts of individual molecules entering and leaving the excitation area in the confocal volume (approximately 10^−15^ L) will result in fluctuations of fluorescence intensity as a function of time. The autocorrelation function, *g(t)*, of the fluorescent molecules in the beam parameter (i.e., radial radii), (*ω_o_*), can be described by the ensemble average of the product of fluorescence intensity at time t, *I(t)*, and that after a delay time *τ*. The function is generally normalized by the square of an average intensity. In addition, the transit time, $\tau_{D}$, which the time required for the fluorescent probe to pass through the confocal volume, can be expressed through the diffusivity or mobility of the molecules (*D*) within the confocal volume as shown in Equation (2).

(2) $$g\left(t\right)=\frac{<\delta I\left(t\right)\cdot \delta I\left(t+\tau \right){>}_{t}}{<I\left(t\right){>}_{t}^{2}}\;,\tau_{D}=\;\frac{\omega_{o}^{2}}{4D}$$

Based on previous research [93,102], the transit time of molecules is determined by the time at which half of the *g(t)* values have been achieved. As the diffusion time is dependent on the types of molecules (i.e., concentration and molecular weight) and its environment, the correlation of fluorescence fluctuation can also be expressed as Equation (3) in order to characterize the fluorescent molecules. The fitting parameter, α shown in Equation (3) in the autocorrelation function, can decipher the type of diffusion of molecules, such as Brownian (*α* = 1) and sub-diffusive/anomalous (0 < *α* < 1).

(3) $$g\left(t\right)=\frac{1}{N}{\left(1+{\left[\frac{t}{\tau_{D}}\right]}^{\alpha }\right)}^{-1}{\left(1+\frac{1}{s^{2}}{\left[\frac{t}{\tau_{D}}\right]}^{\alpha }\right)}^{-1/2}$$

where *N* is the concentration of the molecules and *s* is the structure factor or the axial ratio of the focus [97].

Figure 5a shows that the transit time of SLB has a sluggish diffusion behavior (approximately milliseconds) of more than one order compared to the free dye (Rh6G) in solutions (approximately sub-milliseconds).

The type of diffusion of molecules in the planar membrane can be delineated by mean squared displacement (MSD) of molecules in Equation (4).

(4) $$<r^{2}\left(t\right)>=<{\left(r\left(t\right)–r\left(0\right)\right)}^{2}>=4\;Dt^{\alpha }$$

where <*r*^2^(*t*)> is the mean square displacement (MSD), *r*(*t*) is the position of each molecule in determined time t, *r*(0) is the reference position of each molecule, *α* is anomalous diffusion exponent, and *D* is the diffusion coefficient.

When Brownian diffusion happened (*α* = 1), the lipid molecules performed a two-dimensional random walk in planar membranes that depended on the frictions in surrounding phases or domains [116,117]. Nevertheless, the ideal case for the Brownian diffusion does not often occur in the case of natural membranes and living cells, since the movement of the molecules is occasionally restricted or changed by the environmental heterogeneities (e.g., different lipid phases or rafts) or non-specific interactions of diffusing molecules with other proteins or cellular structures [94,118]. They may have an altered topology during the formation of SLBs on the glass substrates due to the complex compositions in cell membranes. This frequently resulted in sub-diffusive (sluggish) behaviors with 0 < *α* < 1. In cases using free dyes (Rh6G) in the solution, as shown in [119]. Figure 5a, Brownian diffusion (*α* = 1) was confirmed after FCS fitting analysis, where the displacement of dye molecules in the confocal volume was proportional to the measurement time, t. Nevertheless, anomalous sub-diffusive behavior (*α* < 1) was observed in the case of the two SLBs after FCS analysis, as shown in Equation (3), suggesting that the displacement of lipid molecules under the confocal volume exhibited a fractal time dependence. In addition, this further indicated the presence of domains or non-specific interactions with lipids in both SLBs. The dependence of the sub-diffusive behavior was examined on the protein compositions in the SLBs using two different protein contents were exploited with 0.42 mg/mL and 0.84 mg/mL of the RBC related proteins in 10 vol. % of RBC membranes in PBS solutions, respectively. After the extracted RBC membrane, the total protein concentrations in the RBC membranes were confirmed by a typical bicinchoninic acid assay [119]. Figure 5b shows that the increased protein contents in SLBs resulted in a reduced *α* of sub-diffusiveness (*α* < 1), further suggesting that the anomalous sub-diffusion was strongly affected by the factions of proteins in the SLBs. Therefore, this FCS analysis approach can be extended to elucidate unique, non-specific interactions between a variety of membrane-associated proteins and the other domains, because any behavior that cannot hold the Brownian diffusion will perturb the diffusion statistics of the molecules, such as in-situ changes of diffusion coefficients during protein folding or unfolding. Recently, a super-resolution technique has been applied to FCS such that the transit time of the solutes in STED-FCS enables the determination of molecular mobility for observation spot sizes below 50 nm in diameter, [120,121,122] further opening new opportunities to unravel the complex dynamics of native membranes.

## 4. Conclusions

The cell membrane is composed of multiple compartments such as lipids, glycans, and membrane-associated proteins. This review looks at its applications in various fields, such as the biomedical and physical chemistry-associated sciences. In addition to bottom-up techniques with synthetic biomolecules, the rapid development of cell membrane-coated nanocarriers breaks down the limitations involved in the efficiency and compatibility of drug delivery and broadens their biomedical applications due to their inherited multi-functionality. Thanks to the abundant cell sources and its dynamic diversity, there are many opportunities to explore the various new nano-bio interfaces in membrane-coated substrates and multi-length scales, which could provide important clues to the intermolecular forces in cell-mimicking platforms. Further studies could analyze how molecular interactions induce the mobility of individual bio-molecules in different membrane domains and how substrates can be further incorporated with bio-membranes and advanced analytic techniques such as STED-FCSs.

## Figures and Tables

**Table 1:** A summary of the current uses of cell membranes.

Cell Sources	Substrates	Reference	Cell Source	Substrates	Reference
Red Blood Cell membranes	PLGA-NPs	[39,41,45,61]	White Blood Cell membranes	Silica NPs	[50,62]
				ATPEs-Si	[63]
	Au-NPs	[64]		PLGA-NPs	[65]
	Gelatins	[66]		Janus NP	[67]
	Yb^3^+, Er^3^+ and etc.	[68,69]	Platelet membranes	PLGA-NPs	[43,70]
	Iron oxides	[71]	Cancer cell membranes	PLGA-NPs and upcon-version NPs	[42,72]
	Janus particle	[73]	Exosomes	PLGA-NPs	[74]
	Si particles	[62]	Stem cell membranes	gelatin nanogels	[75]
Bacterial membranes (*E. coli*)	Au-NPs	[76,77]		superparamagnetic iron oxide nano-particles (SPIO NPs)	[78]

## Supplementary Materials

Supplementary materials are available online. Video S1: A low surface cover ratio of RBC membranes to Si particles. Video S2: A high surface cover ratio of RBC membranes to Si particles with the successful translocation of the RBC membrane onto the surface of particles.
