# Supplementary material for: The Diverse Range of Possible Cell Membrane Interactions with Substrates: Drug Delivery, Interfaces and Mobility
Source: Molecules. 2017 Dec 11;22(12):2197. doi: 10.3390/molecules22122197 (PMC6149826; doi:10.3390/molecules22122197)
Supplement: Supplementary file 1 [file molecules-22-02197-s001.pptx]

## Slide 1
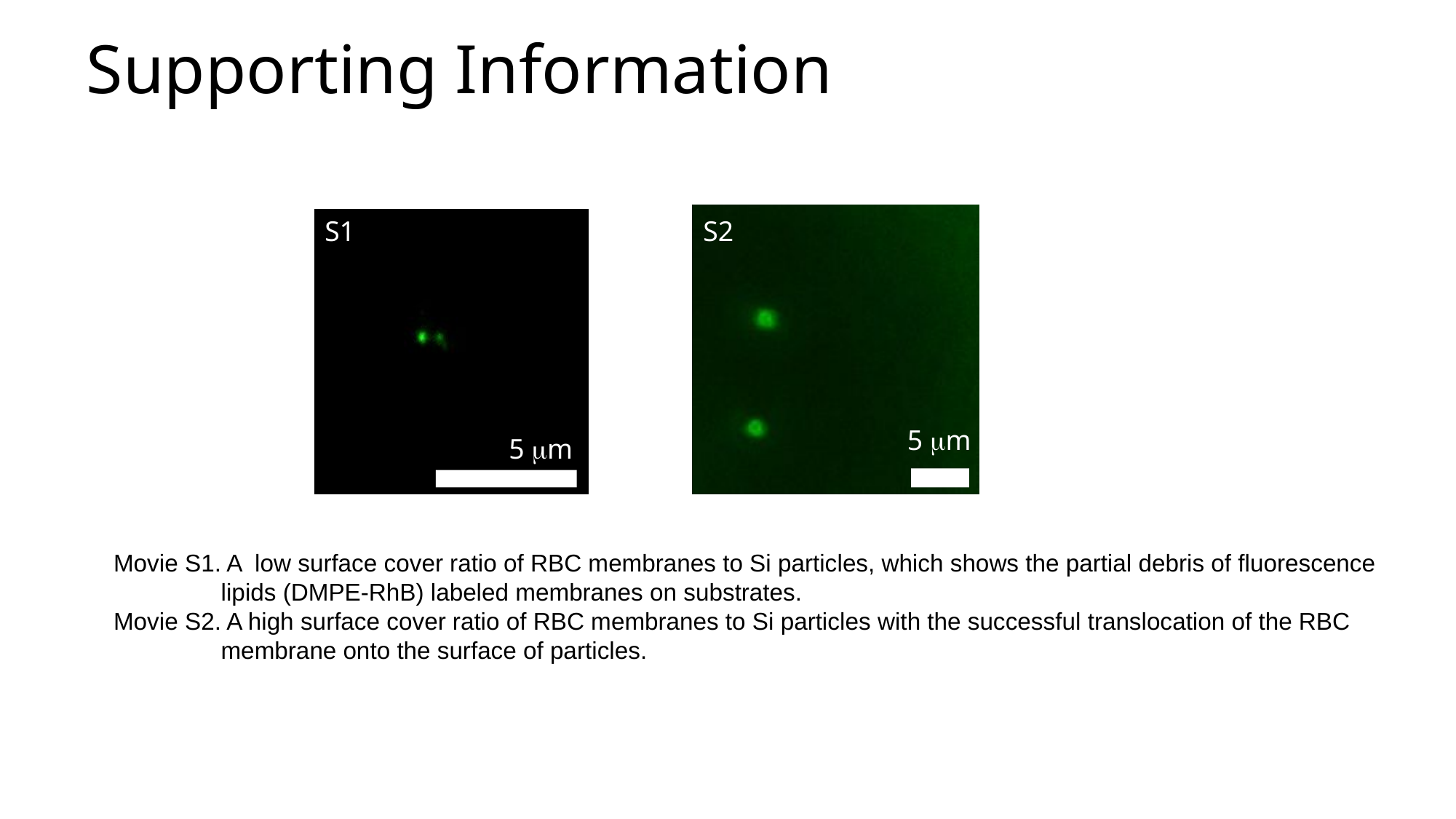

Supporting Information
S2
S1
5 mm
5 mm
Movie S1. A low surface cover ratio of RBC membranes to Si particles, which shows the partial debris of fluorescence
 lipids (DMPE-RhB) labeled membranes on substrates.
Movie S2. A high surface cover ratio of RBC membranes to Si particles with the successful translocation of the RBC
 membrane onto the surface of particles.
